# Legionnaires’ Disease Incidence and Risk Factors, New York, New York, USA, 2002–2011

**DOI:** 10.3201/eid2011.131872

**Published:** 2014-11

**Authors:** Andrea Farnham, Lisa Alleyne, Daniel Cimini, Sharon Balter

**Affiliations:** New York City Department of Health and Mental Hygiene, New York, New York, USA; 1 Current affiliation: University of Zurich, Zurich, Switzerland.

**Keywords:** Legionnaires’ disease, epidemiology, community-acquired infections, New York City, socioeconomic factors, occupations, risk factors, bacteria, Legionella pneumophila, New York, poverty, legionellosis

## Abstract

Incidence of Legionnaires’ disease in the United States is increasing. We reviewed case records to determine the the epidemiology of and risk factors for the 1,449 cases reported to the New York City Department of Health and Mental Hygiene, New York, New York, USA, during 2002–2011. The highest incidence (2.74 cases/100,000 population) occurred in 2009; this incidence was higher than national incidence for that year (1.15 cases/100,000 population). Overall, incidence of Legionnaires’ disease in the city of New York increased 230% from 2002 to 2009 and followed a socioeconomic gradient, with highest incidence occurring in the highest poverty areas. Among patients with community-acquired cases, the probability of working in transportation, repair, protective services, cleaning, or construction was significantly higher for those with Legionnaires’ disease than for the general working population. Further studies are required to clarify whether neighborhood-level poverty and work in some occupations represent risk factors for this disease.

## Abstract 2

Living in low-income areas and working in certain occupations may increase risk.

Legionnaires’ disease, a bacterial infection caused primarily by the species *Legionella pneumophila*, was initially recognized as the cause of a 1976 outbreak of respiratory disease that resulted in 221 cases of illness, primarily among attendees of an American Legion convention in Philadelphia (*1*). In that outbreak, 34 people died, catapulting the previously unidentified disease to national attention (*1*–*4*). Infection with *Legionella* spp. is now classified into 2 clinically distinct diseases, Pontiac fever and Legionnaires’ disease; Pontiac fever is a milder illness that does not involve pneumonia (*2*). 

An estimated 8,000–18,000 persons are hospitalized for legionellosis each year in the United States; ≈5%–30% of case-patients die (*2*,*5*). During the 2000s, cases of legionellosis in the United States reported to the Centers for Disease Control and Prevention increased 279%, from 1,110 in 2000 to 4,202 in 2011. During the same period, the national incidence of legionellosis increased 249%, from 0.39 per 100,000 persons in 2000 to 1.36 per 100,000 persons in 2011 (*6*,*7*). 

Most *Legionella* species live in water, and transmission to humans occurs through inhalation of small water droplets in which the pathogen is aerosolized or by aspiration of contaminated water into the lungs (*2*,*8*). Known host risk factors for legionellosis are smoking, chronic obstructive pulmonary disease, diabetes, immune system compromise, older age (>50 years), and receipt of a transplant or chemotherapy (*9*). Environmental risk factors associated with legionellosis outbreaks are travel, residence in a health care facility, and proximity to cooling towers, whirlpool spas, decorative fountains, and grocery produce misters (*2*,*6*,*10*,*11*). However, only limited studies have been done regarding socioeconomic and occupational risk factors for community-acquired cases; some studies have identified driving as a potential occupational risk factor (*12*,*13*).

To describe the epidemiology of Legionnaires’ disease in New York, New York, we analyzed surveillance data for 2002–2011. In addition to overall incidence, we measured the associations between acquisition of *Legionella* infection and socioeconomic and occupational groups.

## Materials and Methods

### Routine Surveillance Data Collection

The New York City (NYC) Health Code requires providers and laboratories to report all cases of legionellosis in city residents to the NYC Department of Health and Mental Hygiene (DOHMH); these reports include positive results for *Legionella* in cultures, urine antigen tests, direct fluorescent antibody stains, and serologic testing. In addition, all *Legionella*-positive cultures are required to be sent to either the DOHMH Public Health Laboratory or the New York State Department of Health at Wadsworth Center for confirmation, speciation, and serogrouping. For this analysis, residents of the city of New York who had confirmed legionellosis during 2002–2011 were identified by using the Council of State and Territorial Epidemiologists criteria for confirmed cases (*14*). These criteria include radiographic or clinical pneumonia and laboratory diagnosis made by urinary antigen, culture, or 4-fold rise in *L. pneumophila* serum antibody titer. Three cases of Pontiac fever were found during 2002–2011 but were excluded from this study; all legionellosis cases we analyzed were classified as Legionnaires’ disease.

### Case Investigation

DOHMH investigates all urine antigen, culture, direct fluorescent antibody stain, or nucleic acid assay results positive for *Legionella* and all reports of 4-fold or greater rise in antibody titers between acute- and convalescent-phase serum specimens. Because single reports of elevated *Legionella* serum antibody titers are not diagnostic for legionellosis, those reports are investigated on a case-by-case basis. For the investigations, information from medical charts is abstracted, and patients undergo a standardized interview. Data collected are patient sex, age, race/ethnicity, pre-existing medical conditions, occupation, nights away from home, recreational water exposures, and other risk factors for acquiring *Legionella* infection.

All case-patients or case-patient proxies were asked about work, nights away from home, visits to and stays in health care facilities, exposure to water aerosols, and other possible exposures during the 14 days before symptom onset. For this analysis, cases were considered to be definitely health care facility–associated if the case-patient resided in a hospital or nursing home for the entire 10 days (for 2002–2007) or the entire 14 days (for 2008–2011) before onset. (This change in criteria was made in 2008 in consultation with the New York State Department of Health in light of the consensus at that time that the incubation period was 2–14 days. However, NYC DOHMH has since returned to a standard 2–10 day incubation period for this determination.) Cases were considered possibly health care facility–associated if the case-patient resided in a hospital or nursing home for part of the 2–9 days (for 2002–2007) or 2–13 days (for 2008–2011) before onset. All other cases were considered community acquired. 

Death data were recorded by whether the case-patient had died at the time the investigation was closed. Investigations for confirmed cases are considered closed when diagnosis is confirmed and the patient or proxy interview is completed or it is determined that the interview cannot be completed. Death classification may therefore not have included some case-patients who died from legionellosis after the investigation was closed and may have included some who died of causes other than legionellosis.

### Data Sources

Intercensal population estimates for 2002–2009 were produced by DOHMH on the basis of the US Census Bureau Population Estimate Program and housing unit data obtained from the NYC Department of City Planning, available as of November 2012 (*15*). For 2010 and 2011, 2010 US Census data were used.

The Community Health Survey, a yearly cross-sectional telephone survey conducted by DOHMH that provides citywide public health surveillance data (*15*), was used to estimate the prevalence of diabetes in the general population. The American Community Survey, a yearly demographic survey conducted by the US Census Bureau (*16*), was used to calculate population denominators for occupational data. Occupational data collected during case-patient interviews were used to categorize case-patients into American Community Survey–defined occupational classifications.

Neighborhood-level poverty was assessed by using census tract poverty data provided by the US Census. Neighborhood-level poverty was defined as the percentage of residents with household incomes <100% of the federal poverty level on the basis of US Census data from 2000. Census tracts were classified into 6 poverty level categories: very low, <5%; low, 5%–9%; medium, 10%–19%; high, 20%–29%; very high, 30%–39%; and highest, >40%.

### Statistical Analyses

Crude and age-adjusted population-based incidence rates were calculated, with age-adjustments standardized to the US Census 2000 population. Relative risks and their 95% CIs were used to compare the demographic characteristics of legionellosis case-patients with those of the general population of the city of New York. Student *t*-tests were used to compare average incidences between 2 population subgroups. Logistic regression was used to compare death outcomes across sex and acquisition setting while adjusting for age. 

Case-patient addresses were geolocated to census tracts by using ArcMap 10 (http://www.esri.com/software/arcgis). Because health care facility–associated cases may be associated with different risk factor patterns, we restricted occupational and neighborhood-level poverty analyses to community-acquired cases. To assess the relationship between race/ethnicity and socioeconomic status, we calculated crude and age-adjusted legionellosis incidence by race/ethnicity in each neighborhood-level poverty group. All analyses were conducted by using SAS version 9.2 (SAS Institute Inc., Cary, NC, USA). Results were considered statistically significant at p<0.05.

## Results

### Cases

**Figure 1:**
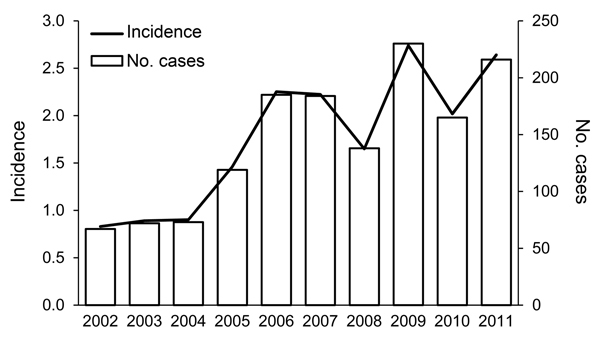
Annual number and incidence (no. cases/100,000 population) of Legionnaires’ disease cases, New York, New York, USA, 2002–2011.

In the city of New York, a total of 1,449 confirmed legionellosis cases were reported through routine surveillance for January 1, 2002–December 31, 2011. Cases were predominantly diagnosed by urinary antigen testing (n = 1,409, 97.2%); 5.7% (n = 82) of all cases were confirmed by culture. The method of diagnosis remained relatively constant over the study period. The crude incidence rate of legionellosis increased from 0.83 cases/100,000 population in 2002 to the highest incidence of 2.74 cases/100,000 population in 2009, a 230% increase (Figure 1). In 2010 and 2011, crude incidence rates remained high at 2.02 and 2.64 cases/100,000 population, respectively. Average yearly incidence for 2002–2011 was 1.75 cases/100,000 population. 

Incidence of legionellosis increased in all age groups during the analysis period, but the largest increase (826%) was for the 70–79 age group from 2002 to 2010. Incidence of community-acquired cases was consistently higher during the summer and early fall months; 71.6% (n = 1,038) of cases were diagnosed during June–October.

### Demographic Variables

**Figure 2:**
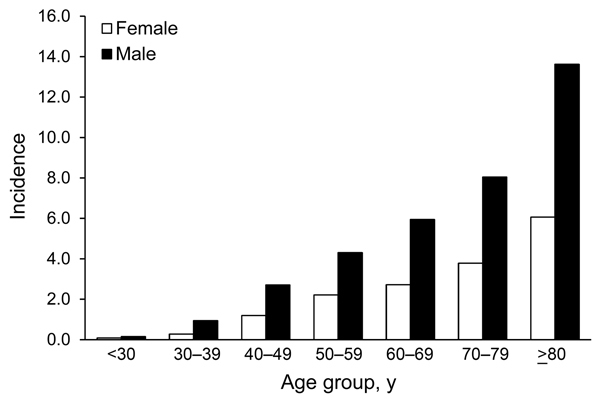
Legionnaires’ disease incidence (no. cases/100,000 population) by sex and age group, New York, New York, USA, 2002–2011.

The average incidence of legionellosis was higher for the male population than for the female population (2.29 cases/100,000 population vs. 1.26 cases/100,000 population; p = 0.0004) and was higher for older age groups (Figure 2). Median case-patient age was 61.0 years (mean 61.8 years, range 9 months to 103 years), and overall incidence rates increased for each age group, with the highest incidence among those >80 years of age (Figure 2). Cases were predominately acquired in the community (88.3%, n = 1,279) rather than definitely (7.0%, n = 102) or possibly (4.7%, n = 68) acquired in a health care facility.

Race/ethnicity was unknown for 279 (19%) case-patients. Average incidence per year for non-Hispanic blacks (2.15 cases/100,000 population) was higher than that for non-Hispanic whites (1.56 cases/100,000 population; p = 0.13) and significantly higher than that for Hispanics (1.02 cases/100,000 population; p = 0.003) or Asian/Pacific Islanders (0.41 cases/100,000 population; p = 0.0004). 

### Deaths

Overall, 12.8% (185/1,449) of legionellosis case-patients died before the DOHMH case investigation was closed. Death rates were significantly higher for case-patients with definitely health care facility–associated infections than for those with community-acquired infections (odds ratio [OR] 4.78, 95% CI 3.07–7.46). Overall, 35.3% (36/102) of case-patients with definitely health care facility–associated infections died, compared with 10.2% (131/1,279) of those with community-acquired infections. The crude odds for death for women were 1.48 times that for men for community-acquired cases (95% CI 1.09–2.02; p = 0.02), but after adjusting for age, the odds for death were not significantly different between men and women (OR 1.33, 95% CI 0.97–1.83; p = 0.07).

### Medical Risk Factors

Of the 1,449 legionellosis cases, 1,278 (88.2%) patients had >1 underlying medical condition that was a known risk factor for legionellosis (*9*). Current or past smoking (n = 879, 60.7%) and diabetes mellitus (n = 506, 34.9%) were the most frequently reported underlying conditions. After stratifying patients by age, the risk for diabetes was higher for persons with legionellosis than for the general population in every age category; the risk for diabetes was 1.9 times higher for legionellosis case-patients >65 years of age, 2.5 times higher for those 45–64 years of age, 5.7 times higher for those 25–44 years of age, and 6.3 times higher for those 18–24 years of age (Table 1).

**Table 1:** Most common underlying medical conditions among Legionnaires’ disease case-patients, New York, New York, USA, 2002–2011*

Condition	No. (%) case-patients	Relative risk (95% CI)†
Smoking history	879 (60.7)	
COPD	223 (15.4)	
Cancer	215 (14.8)	
Diabetes		
Overall	506 (34.9)	
By age group, y†		
18–24	1 (7.7)	6.3 (0.95–41.10)
25–44	31 (15.2)	5.7 (4.1–7.9)
45–64	208 (34.1)	2.5 (2.2–2.8)
>65	264 (42.8)	1.9 (1.7–2.0)

*n = 1,449. COPD, chronic obstructive pulmonary disease. †For case-patients with diabetes only. Population denominators for diabetes prevalence are from the American Community Survey. Relative risks for children age <18 y (2/5 case-patients <18 had diabetes) were not calculated because population denominators were not available for this age group.

### Neighborhood Poverty Level

 To assess neighborhood poverty level, we restricted our analysis to community-acquired cases. Of the 1,279 community-aqcuired cases, 1,261 (98.6%) could be geolocated. After patient age was adjusted for, the incidence of legionellosis for community-acquired cases followed a gradient; incidence in the highest poverty areas (3.0 average yearly cases/100,000 population) was 2.5 times higher than that for the lowest poverty areas (1.2 average yearly cases/100,000 population) (Table 2). As shown in Table 3, the same gradient existed within each racial/ethnic group, with the highest incidence of disease in the highest poverty group. However, after age adjustment, rates of legionellosis among black non-Hispanics remained higher than those for other race/ethnic groups in each poverty group.

**Table 2:** Rates of community-acquired Legionnaires’ disease by census tract poverty level, New York, New York, USA, 2002–2011*

Census tract poverty level†	No. cases	Rate/100,000 population	
		Crude	Age-adjusted‡
Very low	80	1.4	1.2
Low	229	1.5	1.4
Medium	298	1.3	1.4
High	247	1.4	1.6
Very high	196	1.9	2.2
Highest	211	2.3	3.0
Total	1,261	1.6	

*Eighteen community-acquired cases could not be geolocated to a census tract and were excluded from the total case count. †By percentage of residents with household incomes <100% of the federal poverty level according to 2000 U.S. Census data: very low, <5%; low, 5%–9%; medium, 10%–19%; high, 20%–29%; very high, 30%–39%; highest, >40%. ‡Age-adjustment calculations were based on 2000 U.S. Census standard population.

**Table 3:** Sociodemographic characteristics of persons with community-acquired Legionnaires’ disease, New York, New York, USA, 2002–2011*

Poverty level† and race/ethnicity	Cumulative no. cases	Average annual incidence/100,000 population	
		Crude	Age-adjusted‡
Very low			
White non-Hispanic	53	1.3	0.92
Black non-Hispanic	9	1.9	2.0
Hispanic	4	1.0	1.4
Asian/Native Hawaiian/Pacific Islander/American Indian non-Hispanic	4	0.83	1.5
Other/multirace non-Hispanic	0	0	0
Unknown	10		
Low			
White non-Hispanic	130	1.4	1.1
Black non-Hispanic	34	1.5	1.6
Hispanic	13	0.79	1.0
Asian/Native Hawaiian/Pacific Islander/American Indian non-Hispanic	11	0.74	1.1
Other/multirace non-Hispanic	3	0.62	1.2
Unknown	38		
Medium			
White non-Hispanic	112	1.3	0.98
Black non-Hispanic	76	1.7	1.8
Hispanic	45	0.92	1.2
Asian/Native Hawaiian/Pacific Islander/American Indian non-Hispanic	7	0.23	0.29
Other/multirace non-Hispanic	4	0.38	0.50
Unknown	54		
High			
White non-Hispanic	50	1.3	0.94
Black non-Hispanic	88	1.7	1.9
Hispanic	40	0.75	1.1
Asian/Native Hawaiian/Pacific Islander/American Indian non-Hispanic	6	0.31	0.39
Other/multirace non-Hispanic	3	0.43	0.60
Unknown	60		
Very high			
White non-Hispanic	24	2.3	1.8
Black non-Hispanic	78	2.2	2.5
Hispanic	48	1.0	1.4
Asian/Native Hawaiian/Pacific Islander/American Indian non-Hispanic	8	0.98	1.1
Other/multirace non-Hispanic	3	1.1	1.5
Unknown	35		
Highest			
White non-Hispanic	20	2.6	2.6
Black non-Hispanic	91	2.7	3.3
Hispanic	55	1.2	1.7
Asian/Native Hawaiian/Pacific Islander/American Indian non-Hispanic	0	0	0
Other/multirace non-Hispanic	1	0.45	0.87
Unknown	44		
Total	1,261		

*Eighteen cases could not be geolocated to a census tract and were excluded from the total case count. †By percentage of residents with household incomes <100% of the federal poverty level according to 2000 U.S. Census data: very low, <5%; low, 5%–9%; medium, 10%–19%; high, 20%–29%; very high, 30%–39%; highest, >40%. ‡Age-adjustment calculations were based on 2000 U.S. Census standard population.

### Occupation

Among the 1,279 community-acquired legionellosis cases, 375 (29.3%) case-patients reported working in the 2 weeks before disease onset. The average and median age of case-patients who worked was 53.0 years, and the average age of case-patients who did not work was 64.9 years (median 65.0 years), a mean difference of 11.9 years (95% CI 9.9–13.8 years; p<0.0001). A higher percentage of working case-patients were male than female (71.5% vs. 59.1%; p<0.0001). Compared with the general population, legionellosis case-patients were significantly more likely to work in transportation (crude relative risk [RR] 2.36, 95% CI 1.82–3.06), repair (crude RR 1.86, 95% CI 1.11–3.11), protective services (crude RR 1.77, 95% CI 1.15–2.71), cleaning services (crude RR 1.54, 95% CI 1.07–2.22), or construction (crude RR 1.49, 95% CI 1.03–2.16) (Table 4).

**Table 4:** Incidence of and risk for Legionnaires’ disease by case-patient occupation, New York, New York, USA, 2002–2011

Occupational category	No. (%) working case-patients	% Total working population*	Mean crude annual disease incidence†	Crude relative risk (95% CI)‡
Transportation	49 (13.1)	5.5	1.9	2.36 (1.82–3.06)
Repair	14 (3.7)	2.0	1.5	1.86 (1.11–3.11)
Protection	20 (5.3)	3.0	1.4	1.77 (1.15–2.71)
Cleaning	27 (7.2)	4.7	1.3	1.54 (1.07–2.22)
Construction	26 (6.9)	4.7	1.2	1.49 (1.03–2.16)
Service	24 (6.4)	5.0	1.0	1.28 (0.87–1.89)
Legal	10 (2.7)	2.1	1.0	1.27 (0.69–2.34)
Office	59 (15.7)	15.0	0.9	1.05 (0.83–1.33)
Entertainment	17 (4.5)	4.6	0.8	0.98 (0.61–1.55)
Production	14 (3.7)	3.8	0.8	0.99 (0.59–1.65)
Counsel	7 (1.9)	2.1	0.7	0.91 (0.43–1.89)
Finance	8 (2.1)	2.7	0.6	0.78 (0.39–1.55)
Medical	12 (3.2)	4.2	0.6	0.76 (0.43–1.32)
Food	16 (4.3)	5.3	0.7	0.80 (0.50–1.29)
Health	10 (2.7)	4.1	0.5	0.66 (0.36–1.21)
Sales	27 (7.2)	11.3	0.5	0.64 (0.44–0.92)
Engineering	2 (0.5)	0.9	0.5	0.58 (0.15–2.33)
Management	11 (2.9)	8.1	0.3	0.36 (0.20–0.65)
Education	6 (1.6)	5.9	0.2	0.27 (0.12–0.60)
Computer	1 (0.3)	2.0	0.1	0.13 (0.02–0.95)
Uncategorized/missing	15 (4.0)	0.0	NA	
Total working	375 (100.0)	100.0	0.8	

*Percentage of total city population working in occupational category (American Community Survey occupational survey). †Per 100,000 population, based on number of persons working in occupational category, according to 2005–2009 American Community Survey population data. ‡Comparison of risk for being in each occupational category for working case-patients versus the general working population.

## Discussion

Surveillance data show a sustained increase in legionellosis incidence in the city of New York during 2002–2011, a trend that is also reflected in data nationwide (*6**,**7**,**17*). During this period, legionellosis incidence in New York was highest in 2009 (2.74 cases/100,000 population), higher than overall US incidence in 2009 (1.5 cases/100,000 population) (*6*). For 2011, the last year of the study, legionellosis incidence in New York was 2.64 cases/100,000 population, higher than US overall incidence in 2011 (1.36 cases/100,000 population) (*7*). 

Urinary antigen testing remained the primary method of legionellosis diagnosis during the study period, indicating that the increases are most likely not an artifact of changes in diagnostics. However, it is possible that increased awareness of the disease among clinicians led to increased testing over time. The overall epidemiology of legionellosis cases in New York is similar to that reported elsewhere, with higher incidence among men and in older age groups (*6**,**7**,**11*). In addition, several host, socioeconomic, and occupational factors were significantly associated with the risk of acquiring *Legionella* infection among case-patients in New York.

More than 88% of legionellosis case-patients in this study had >1 underlying medical condition that is recognized as a risk factor for infection, a rate higher than that reported for legionellosis populations in Italy and France (*18*,*19*). A smoking history and diabetes were the most commonly reported risk factors for legionellosis patients in New York, a result that has also been seen elsewhere in the United States (*8*). Prevalence of diabetes among legionellosis patients in New York was higher than that for the general population of the city in every age category for which data were available, although the number of legionellosis cases in the 18–24-year-old group was small (n = 13), and prevalence of diabetes did not achieve statistical significance in this group. The prevalence of legionellosis case-patients with diabetes (34.9%) was also higher than that reported among persons with legionellosis in France (*19*). Given the available data for 2002–2011, we were unable to determine percentages of type 1 and type 2 diabetes among the case-patients with diabetes; because type 1 diabetes is not currently preventable, this limitation restricts the ability to make inferences about whether diabetes may be a preventable risk factor for acquisition of *Legionella* infection.

We found that socioeconomic factors were associated with increased incidence of legionellosis. The data show a distinct gradient in incidence according to neighborhood-level poverty, ranging from 3.0 average yearly cases/100,000 population in the highest-poverty areas to 1.2 average yearly cases/100,000 population in the lowest-poverty areas. Because >98% of community-acquired legionellosis cases in this study were geocoded to a census tract, these results are unlikely to be biased. Legionellosis incidence also varied by racial and ethnic groups, with the highest incidence among non-Hispanic blacks, a finding that was also noted in a review of nationwide legionellosis cases reported to the Centers for Disease Control and Prevention during 2000–2009 (*6*). Surveillance data included in our study indicate substantial disparities in incidence of legionellosis by race/ethnicity and socioeconomic factors. The association between race/ethnicity and neighborhood-level poverty suggests that socioeconomic factors may contribute to the disparity in incidence by race/ethnicity. It remains unclear whether the disparities in legionellosis incidence arise from differences in neighborhood environments, such as building construction and maintenance; from differences in access to preventive health care services that could potentially improve conditions such as diabetes; or from differences in the population itself, such as increased levels of diabetes and other host risk factors (*20*,*21*).

We also found that certain occupations might be associated with increased risk for community-acquired legionellosis. Occupations that may involve working with machinery or being outdoors (e.g., transportation, construction, manufacturing) were associated with increased risk of acquiring *Legionella* infection. The increased risk associated with cleaning and janitorial work may involve greater exposure to aerosolized water in the air, perhaps through contact with plumbing systems. The increased risk associated with being a protective services worker (e.g., police officer, crossing guard, security guard) has a less clear causal pathway but may involve a higher exposure to aerosolized water in the outdoors. Other studies in the United States regarding occupational risks for acquiring *Legionella* infection are lacking, making it difficult to determine whether these results are consistent with trends observed elsewhere. However, the results we found for transportation workers are consistent with studies regarding increased risk of *Legionella* infection in England, the Netherlands, and Japan (*12*,*22*,*23*).

Our study has several limitations. First, legionellosis is likely underdiagnosed, as has been documented elsewhere (*24*). Second, we did not have race/ethnicity data for 19% of the case-patients in our study; if the rate of missing data differs across race/ethnicity groups, the estimates of incidence by race/ethnicity may be biased and could underestimate the true incidence of disease in at least some groups. Third, during the analysis period, we identified some health care facility–associated outbreaks and some community-associated clusters that may or may not have been outbreaks involving a point source exposure. Although most cases included in the review period were sporadic rather than outbreak-related, the inclusion of both types of cases in this review obscures possible differences between sporadic and outbreak-related cases in terms of exposure and host-patient factors. Fourth, travel-associated cases were not excluded from this analysis. Surveillance staff did ask case-patients if they spent a night away from home during the incubation period, but we cannot determine whether the case was “travel-associated” on the basis of this question because it would have included respondents who did not travel outside the city. 

Fifth, although the data indicate that an association between occupation and socioeconomic status is likely, we were unable to adjust for this possibility because of small numbers. The low percentage of case-patients with community-acquired legionellosis who reported working in the 2 weeks before illness onset (n = 375, 29.3%) probably reflects the role of advanced age (i.e., many who acquire this infection are retired), medical conditions, or both as risk factors for disease. If underdiagnosis is higher for certain occupational groups, possibly because of socioeconomic factors and differences in access to health care or access to diagnostic tests for *Legionella* infection, a decreased association between certain occupations and risk for acquiring infection may result. Some correlation likely exists between occupational group and socioeconomic status, but it is difficult to make assumptions about individual income levels on the basis of occupational category. Another limitation is the lack of exact measurement of the occupational risk factors for each individual; this study makes assumptions about the average levels of exposures in each occupational category that may not hold true for individual patients within each category, especially because the occupational categorization was done after data collection. In addition, because of the small numbers within some occupational categories and the nature of surveillance data, these analyses could not be adjusted for confounders that may be related to occupation and risk of acquiring *Legionella* infection. However, among the occupational groups for which there was a univariate association with risk of acquiring *Legionella* infection, the frequency of smoking and frequency of diabetes were not significantly elevated. In view of the limitations of the data, interpretation of these findings should be used primarily for hypothesis generation. More careful measurement of occupational risk factors during routine surveillance may help clarify the causal pathway between occupation and risk of disease.

Despite these limitations, our findings suggest systemic differences in the risk for acquiring *Legionella* infection based on neighborhood-level poverty; these socioeconomic disparities in disease should be of concern to public health policy makers. If environmental issues in high-poverty neighborhoods contribute to the disparity, greater effort may be warranted, for example, on the upkeep of cooling towers and water systems in the buildings in these areas. If occupations such as working in transportation, construction, and plumbing involve increased exposure to *Legionella* and risk for acquiring infection, public health interventions that target these occupational groups (e.g., use of personal protective equipment such as respirators under certain working conditions) may be effective in reducing legionellosis incidence. Future studies are needed to clarify the exact mechanisms by which these host, socioeconomic, and occupational exposures may contribute to *Legionella* infection to help guide public health interventions. Socioeconomic and occupational factors represent important and understudied potential sources of exposure among community-acquired cases of legionellosis.
